# In vitro delivery efficiencies of nebulizers for different breathing patterns

**DOI:** 10.1186/s12938-021-00895-3

**Published:** 2021-06-10

**Authors:** Hyun Mok Park, Kyung Hwa Chang, Sang-Hyub Moon, Bong Joo Park, Sun Kook Yoo, Ki Chang Nam

**Affiliations:** 1 Department of Medical Engineering, Dongguk University College of Medicine, Goyang, South Korea; 2 Graduate Program of Biomedical Engineering, Yonsei University, Seoul, South Korea; 3 Department of Electrical & Biological Physics, Kwangwoon University, Seoul, South Korea; 4 Institute of Biomaterials, Kwangwoon University, Seoul, South Korea; 5 Department of Medical Engineering, Yonsei University College of Medicine, Seoul, South Korea

**Keywords:** Nebulizer, Respiratory simulator, Drug delivery, Dosage, Efficiency

## Abstract

**Background:**

Nebulizers are medical devices that deliver aerosolized medication directly to lungs to treat a variety of respiratory diseases. However, breathing patterns, respiration rates, airway diameters, and amounts of drugs delivered by nebulizers may be respiratory disease dependent.

**Method:**

In this study, we developed a respiratory simulator consisting of an airway model, an artificial lung, a flow sensor, and an aerosol collecting filter. Various breathing patterns were generated using a linear actuator and an air cylinder. We tested six home nebulizers (jet (2), static (2), and vibrating mesh nebulizers (2)). Nebulizers were evaluated under two conditions, that is, for the duration of nebulization and at a constant output 1.3 mL using four breathing patterns, namely, the breathing pattern specified in ISO 27427:2013, normal adult, asthmatic, and COPD.

**Results:**

One of the vibrating mesh nebulizers had the highest dose delivery efficiency. The drug delivery efficiencies of nebulizers were found to depend on breathing patterns.

**Conclusion:**

We suggest a quantitative drug delivery efficiency evaluation method and calculation parameters that include considerations of constant outputs and residual volumes. The study shows output rates and breathing patterns should be considered when the drug delivery efficiencies of nebulizers are evaluated.

## Background

Nebulizers are mainly used to treat and manage patients with respiratory diseases such as asthma, chronic obstructive pulmonary disease (COPD), cystic fibrosis, or pneumonia. Aerosolized medications have the advantage of delivering drugs directly to lungs. Nebulizers can be classified into three types, i.e., jet, ultrasonic, and mesh nebulizers, according to their operating principles [1–3]. Jet nebulizers have large output rates and mass distribution variations that depending on nozzle and compressor combinations, but are noisy, vibrate in use, and have large residual volumes [3–5]. Mesh nebulizers were developed to overcome these limitations and produce aerosols through holes in a mesh or plate. These nebulizers are classified as passive (static) or active (vibrating) based on their operating principles. Static mesh nebulizers use an ultrasonic horn that vibrates a static mesh indirectly, whereas in vibrating mesh nebulizers the mesh is mounted on a vibrating piezoelectric ring [1, 6, 7].

For oral and intravenous drug administrations, drug delivery is obviously 100% [8, 9], but aerosol-based treatment deliveries are only 10–15% efficient based on the amounts of drugs loaded [10, 11]. Nevertheless, because drugs are delivered directly to lungs, the therapeutic effects of aerosol delivery are high, and as a result, nebulizer-based aerosol therapies have the advantages of lower drug doses, rapid onsets, fewer side effects, and convenience over systemic drug delivery [1, 12–16]. However, the amounts of drugs delivered by nebulizers vary considerably [1, 14, 17, 18]. For example, breathing patterns and physiological factors such as airway diameters are disease-type-dependent and can affect drug delivery efficiencies [17, 18]. In addition, particle sizes, output rates, residual volumes, and nebulization times are dependent on nebulizer type. It has been reported that in vivo aerosol delivery from a nebulizer can be estimated by simulating breathing patterns in vitro [19], which is more convenient and reproducible than in vivo testing. Furthermore, comparative studies conducted using respiratory simulators have shown that mesh nebulizers deliver the same amount or more drugs than jet nebulizers [20–23].

The International Organization for Standardization (ISO) 27427:2013 (Anaesthetic and respiratory equipment-Nebulizing systems and components) defines a method for measuring the outputs and output rates of nebulizers [24], but it does not include a method for testing drug delivery efficiency. Currently, there is no standard test for measuring drug delivery efficiency, although several in vivo and in vitro respiratory simulator studies have described potential test methods [3, 17–23, 25–28]. In vivo animal models may not adequately reflect real clinical settings [3], whereas in vitro respiratory simulator testing provides a more convenient means of testing than in vivo methods and provides reproducible results [19].

In this study, we developed a respiratory simulator that meets the volume control requirement of the ventilator standard. Six home nebulizers of three types (jet, static mesh, and vibrating mesh) were tested using an in vitro adult airway model for the duration of nebulization and at a constant output of 1.3 mL using four breathing patterns (ISO 27427:2013, normal adult, asthmatic and COPD patterns).

## Results

### Breathing patterns

**Fig. 1:**
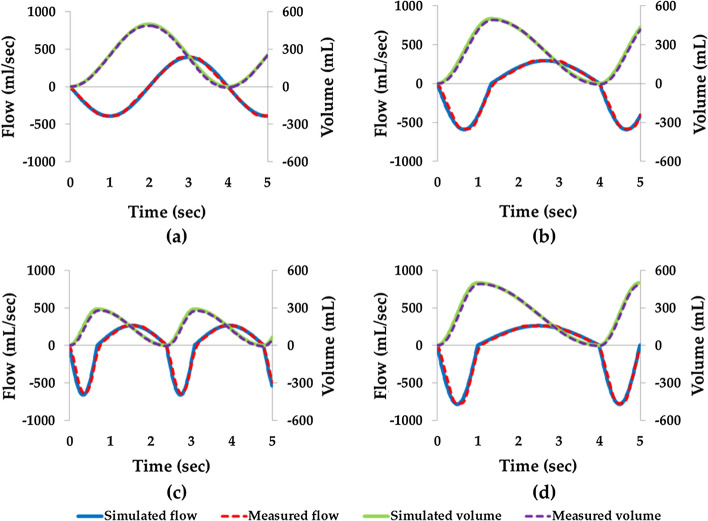
Breathing patterns generated by the respiratory simulator: **a** ISO 27427:2013; **b** normal adult; **c** asthma; and **d** COPD. Simulated flow (blue solid line); measured flow (red dotted line); simulated volume (green solid line); measured volume (purple dotted line)

We generated four breathing patterns using the respiratory simulator, that is, the pattern specified in ISO 27427:2013, normal adult, asthmatic, and COPD patterns (Fig. 1). Simulated breathing flows (blue solid line) and measured flows (red dashed line) were well correlated for all four patterns (ISO 27427:2013: 0.991, normal adult pattern 0.992, asthma 0.988, and COPD: 0.992). Breathing patterns were generated air flows 10 respiratory cycles and mean measured breath volumes were ISO 27427:2013: 492.51 ± 0.24 mL, normal adult: 495.71 ± 3.75 mL, asthma: 284.40 ± 2.34 mL and COPD: 494.02 ± 2.02 mL. The average volume error of breathing patterns was 1.46 ± 0.73%, which met the ISO standard requirement (ISO 80601-2-12:2011), which states that breath volume error should be within ± (5 mL + 10% of the set volume) [29].

### Comparison of delivery efficiencies by breathing patterns

#### Experiment 1

This experiment was designed to evaluate delivery efficiencies in clinical situations. The doses delivered by static and vibrating mesh nebulizers were similar or greater than those delivered by jet nebulizers for all breathing patterns (Table 1). Percentage delivered dosage for loading dose (DD/LD) and percentage delivered dosage for emitted dose (DD/ED) for vibrating mesh nebulizers were similar because they had negligible residual volume. However, the jet and static nebulizers, which had larger residual volumes, showed substantial differences. The vibrating mesh nebulizer VMN-SM1 had the highest delivered dose, DD/LD, and DD/ED. In Experiment 1, DD/ED values were considered inappropriate because of nebulizer output differences, whereas DD/LD comparisons allowed evaluations that included considerations of residual volume (waste). However, DD/LD values were unsuitable for comparing delivery efficiencies because of the different output rates and residual volumes. Therefore, in Experiment 2, we compared nebulizer DD/ED values obtained at constant emitted doses.

**Table 1:** The six nebulizer types used in the study, delivered doses and percentage delivered dosage for loading dose (DD/LD) and emitted dose (DD/ED) values obtained in Experiment 1 (mean ± SD) *$p < 0.05$ versus the JN-PARIr; JN-PARIr: PARI BOY SX + LC SPRINT—red nozzle, JN-PARIb: PARI BOY SX + LC SPRINT—blue nozzle, SMN-U150: NE-U150, SMN-U22: NE-U22, VMN-SM1: NE-SM1 NEPLUS, VMN-SM3: NE-SM3

Breathing	Parameter	Jet		Static mesh		Vibrating mesh	
		JN-PARIr	JN-PARIb	SMN-U22	SMN-U150	VMN-SM1	VMN-SM3
ISO 27427:2013	Delivered dose (mg)	0.403 ± 0.010	0.277 ± 0.014*	0.416 ± 0.005	0.420 ± 0.016	0.589 ± 0.012*	0.389 ± 0.011
	DD/LD (%)	20.17 ± 0.51	13.83 ± 0.70*	20.78 ± 0.24	21.01 ± 0.80	29.48 ± 0.60*	19.45 ± 0.56
	DD/ED (%)	27.61 ± 0.70	20.18 ± 1.02*	21.85 ± 0.25*	26.62 ± 1.02	29.61 ± 0.61*	19.60 ± 0.57*
Normal adult	Delivered dose (mg)	0.251 ± 0.007	0.163 ± 0.010*	0.305 ± 0.024*	0.281 ± 0.012	0.419 ± 0.018*	0.244 ± 0.004
	DD/LD (%)	12.59 ± 0.37	8.13 ± 0.50*	15.25 ± 1.20	14.06 ± 0.61	20.94 ± 0.88*	12.21 ± 0.71
	DD/ED (%)	17.24 ± 0.51	11.86 ± 0.72*	16.02 ± 1.27	17.81 ± 0.77	21.03 ± 0.88*	12.30 ± 0.71*
Asthma	Delivered dose (mg)	0.186 ± 0.022	0.106 ± 0.015*	0.106 ± 0.015*	0.199 ± 0.013	0.353 ± 0.011*	0.157 ± 0.015
	DD/LD (%)	9.28 ± 1.17	5.34 ± 0.76*	5.34 ± 0.76*	9.97 ± 0.68	17.65 ± 0.54*	7.83 ± 0.74
	DD/ED (%)	12.70 ± 1.51	7.79 ± 1.11*	7.79 ± 1.11*	12.62 ± 0.87	17.73 ± 0.54*	7.89 ± 0.75*
COPD	Delivered dose (mg)	0.144 ± 0.010	0.097 ± 0.002*	0.097 ± 0.002*	0.170 ± 0.025	0.259 ± 0.002*	0.137 ± 0.011
	DD/LD (%)	7.22 ± 0.49	4.85 ± 0.11*	4.85 ± 0.11*	8.51 ± 1.26	12.96 ± 0.11*	6.85 ± 0.56
	DD/ED (%)	9.88 ± 0.66	7.07 ± 0.15*	7.07 ± 0.15*	10.79 ± 1.59	13.02 ± 0.11*	6.90 ± 0.56*

**Fig. 2:**
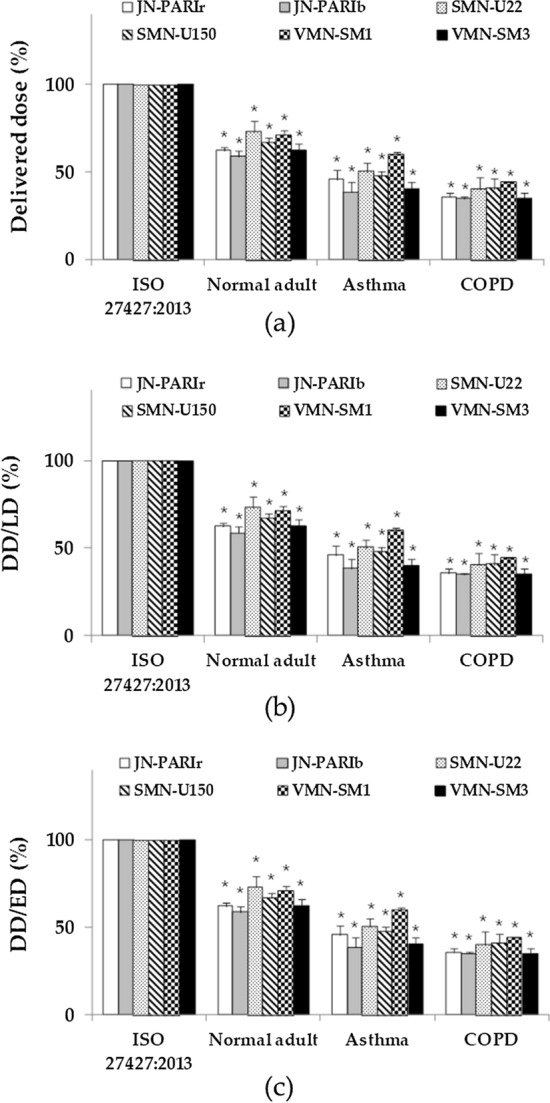
Delivered doses (**a**), percentage delivered dosage for loading dose (DD/LD) (**b**), and dose emitted (DD/ED) (**c**) compared to breathing pattern for ISO 27427:2013 in Experiment 1. JN-PARIr (white); JN-PARIb (gray); SMN-U22 (dots); SMN-U150 (diagonal stripes); VMN-SM1 (checkered); VMN-SM3 (black) (result are means ± SDs), *$p < 0.05$ versus the ISO 27427:2013 breathing pattern

Delivered dose of the nebulizer was different according to the breathing patterns. Compared with ISO 27427:2013, the delivered dose was significantly reduced in the other breathing patterns (Fig. 2). Delivered doses and delivery efficiencies decreased in the order ISO 27427:2013 > normal adult > asthma > COPD. The tidal volume of the asthma pattern was smaller than that of COPD, but delivered doses and delivery efficiencies were higher or similar to COPD due to a higher respiration rate and a lower expiration phase.

#### Experiment 2

Experiment 2 was designed to evaluate the drug solution delivery efficiency quantitatively. To make emitted dose identical, the residual volume remained intentionally even in mesh nebulizer. Therefore DD/LD became lower than in Experiment 1. We compared drug delivery efficiency using the same emitted doses for all six nebulizers (Table 2). The VMN-SM1 nebulizer had a higher DD/ED than the other nebulizers for all breathing patterns.

In Experiment 2, the emitted dose (ED) for all nebulizers was set 1.3 mL to account for the residual volume by JN-PARIb. The ED of each nebulizer was lower than in Experiment 1 except for the JN-PARIb. Delivered doses (DDs) in Experiment 2 reduced similarly by the decreases of ED proportionally compared to Experiment 1, and thus, DD/ED values were similar in Experiments 1 and 2.

**Table 2:** The six nebulizer types used in the study, delivered dose and percentage delivered dosage for loading dose (DD/LD) and emitted dose (DD/ED) values obtained in Experiment 2 (mean $\pm$ SD) *$p < 0.05$ versus the JN-PARIr

Breathing	Parameter	Jet		Static mesh		Vibrating mesh	
		JN-PARIr	JN-PARIb	SMN-U22	SMN-U150	VMN-SM1	VMN-SM3
ISO 27427:2013	Delivered dose (mg)	0.312 $\pm$ 0.037	0.256 $\pm$ 0.008*	0.281 $\pm$ 0.008	0.353 $\pm$ 0.012	0.417 $\pm$ 0.015*	0.251 $\pm$ 0.028*
	DD/LD (%)	15.60 $\pm$ 1.87	12.80 $\pm$ 0.41*	14.06 $\pm$ 0.38	17.68 $\pm$ 0.59	20.86 $\pm$ 0.74*	12.58 $\pm$ 1.37*
	DD/ED (%)	24.01 $\pm$ 2.87	19.70 $\pm$ 0.63*	21.63 $\pm$ 0.59	27.20 $\pm$ 0.91	32.09 $\pm$ 1.14*	19.35 $\pm$ 2.11*
Normal adult	Delivered dose (mg)	0.197 $\pm$ 0.020	0.147 $\pm$ 0.004*	0.171 $\pm$ 0.009	0.210 $\pm$ 0.008	0.247 $\pm$ 0.013*	0.156 $\pm$ 0.008*
	DD/LD (%)	9.84 $\pm$ 1.02	7.35 $\pm$ 0.18*	8.53 $\pm$ 0.46	10.50 $\pm$ 0.38	12.38 $\pm$ 0.64*	7.81 $\pm$ 0.42*
	DD/ED (%)	15.14 $\pm$ 1.57	11.31 $\pm$ 0.28*	13.12 $\pm$ 0.71	16.16 $\pm$ 0.59	19.04 $\pm$ 0.98*	12.01 $\pm$ 0.65*
Asthma	Delivered dose (mg)	0.137 $\pm$ 0.020	0.092 $\pm$ 0.010*	0.111 $\pm$ 0.004*	0.155 $\pm$ 0.002	0.217 $\pm$ 0.007*	0.112 $\pm$ 0.011
	DD/LD (%)	6.83 $\pm$ 1.04	4.63 $\pm$ 0.48*	5.52 $\pm$ 0.23*	7.75 $\pm$ 0.10	10.88 $\pm$ 0.36*	5.61 $\pm$ 0.57
	DD/ED (%)	10.51 $\pm$ 1.59	7.13 $\pm$ 0.73*	8.49 $\pm$ 0.35*	11.92 $\pm$ 0.15	16.74 $\pm$ 0.56*	8.63 $\pm$ 0.88
COPD	Delivered dose (mg)	0.130 $\pm$ 0.012	0.088 $\pm$ 0.010*	0.108 $\pm$ 0.011	0.114 $\pm$ 0.008	0.176 $\pm$ 0.028*	0.093 $\pm$ 0.008*
	DD/LD (%)	6.48 $\pm$ 0.60	4.42 $\pm$ 0.49*	5.56 $\pm$ 0.21	5.73 $\pm$ 0.41	8.84 $\pm$ 1.42*	4.66 $\pm$ 0.38*
	DD/ED (%)	9.97 $\pm$ 0.92	6.80 $\pm$ 0.74*	8.56 $\pm$ 0.32	8.82 $\pm$ 0.63	13.59 $\pm$ 2.19	7.18 $\pm$ 0.59*

**Fig. 3:**
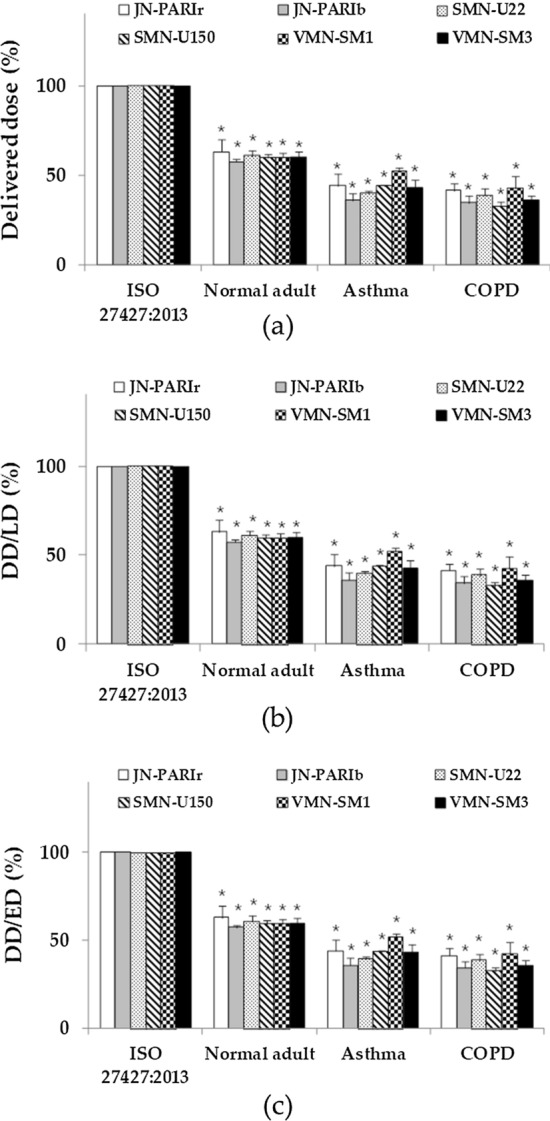
Delivered doses (**a**), percentage delivered dosage for loading dose (DD/LD) (**b**), and dose emitted (DD/ED) (**c**) compared to breathing pattern for ISO 27427:2013 in Experiment 2. JN-PARIr (white); JN-PARIb (gray); SMN-U22 (dots); SMN-U150 (diagonals stripes); VMN-SM1 (checkered); VMN-SM3 (black) (result are means ± SDs), *$p < 0.05$ versus the ISO 27427:2013 breathing pattern

Delivered doses and delivery efficiencies for different breathing patterns followed the order ISO 27427:2013 > normal adult > asthma > COPD, which was the same as that observed in Experiment 1 (Fig. 3).

## Discussion

Nebulizers are usually used at home or in hospitals to treat respiratory diseases. In this study, we developed a respiratory simulator that enables nebulizer drug delivery testing quantitatively. Six home nebulizers (JN-PARIr, JN-PARIb, SMN-U22, SMN-U150, VMN-SM1, and VMN-SM3) were tested using the developed respiratory simulator. Other studies have shown that delivered doses are affected by particle size, breathing patterns, and airway models [17, 18, 26, 30–33]. Therefore, there is a need for a respiratory simulator that includes an airway model capable of generating various stable breathing patterns. The developed respiratory simulator generated four breathing patterns including that mentioned in ISO 27427:2013.

It is difficult to compare the drug delivery efficiencies of different nebulizer types because drug outputs vary considerably for each nebulizer. Thus, we investigated the drug delivery efficiencies of the six nebulizers using two conditions: Experiment 1 (the duration of nebulization) and Experiment 2 (constant output of 1.3 mL). In Experiment 1, the jet nebulizers had smaller or similar drug deliveries than the mesh nebulizers (Table 1), which concurs with previous reports [20–23, 25]. Notably, it has also been reported delivered doses are particle size, residual volume, and output rate-dependent even for a single nebulizer type [4, 5, 34]. The delivered dosesby the VMN-SM1 (a vibrating mesh nebulizer) were higher than those of other mesh nebulizers. In a previous study, in which salbutamol was collected without an airway model using the ISO 27427:2013 breathing pattern, DD/LD value was ~ 41.16% for SMN-U22 nebulizers [28]. However, the calculated percentage delivered dose for the fine particle fraction ($\hbox {FPF} < 5 {\upmu}\hbox {m}$) was 15.68% [28]. In the present study, based on the amount of drug collected by the filter in an airway containing model, DD/LD was ~ 20.8%. Delivered dose may depend on particle size due to the effect of the airway [26, 30–33]. In this study, we compared the drug delivery efficiencies using an airway model rather than calculating the FPF. In nebulizers with a large residual volume, the difference between DD/LD and DD/ED value is high, which reflects drug wastage. DD/LD valuesare applicable when evaluating drug usage and considering residual volume. In Experiment 2, we compared drug delivery efficiencies (DD/ED) by setting the outputs of all nebulizers to 1.3 mL. VMN-SM1 had a higher delivered dose because its DD/ED values were higher than those of the JN-PARIr nebulizer (Table 2).

In Experiments 1 and 2, it was observed that delivered doses depended on breathing patterns (Figs. 2 and 3). In particular, delivered doses in diseased states were lower than for the ISO 27427:2013 breathing pattern in Experiments 1 and 2, which agrees with a previous report [17, 18]. The inspiration phase of the Inspiration:Expiration (I:E ratio) was longest for the ISO 27427:2013 pattern and shortest for COPD, which suggests delivered dose might depend on the I:E ratio. Also, the amount of drug delivered might depend on tidal volume and respiration rate. A longer inspiration phase is related to a higher delivered dose across all breathing patterns, whereas a longer expiration phase reduced the delivered dose [17, 18]. ISO 27427:2013 defines only one breathing pattern [24], and it has been reported that data obtained using one breathing pattern may result in inappropriate clinical applications [17]. Our results agree with these findings, which suggests a test is required that better mimics patient breathing patterns.

This study was performed using a respiratory simulator to determine nebulizer drug delivery efficiencies in vitro under conditions that are representative of actual clinical environments. In reality, breathing patterns are usually irregular, which can cause inconsistent drug delivery [20]. On the other hand, respiratory simulators produce consistent breathing patterns and specific respiratory diseases [35]. However, we suspect our delivered dose results may have been overestimations because the airway model used did not mimic disease conditions exactly. Furthermore, drug delivery characteristics are probably different for adults and children [20] and depend on drug characteristics [36–38] and nebulizer interfaces [1, 20]. Further research is needed to better match breathing patterns with those observed clinically, to design airway models suitable for respiratory diseases, and to determine the effects of different drugs and nebulizer interfaces on drug delivery.

## Conclusion

We developed a respiratory simulator and compared the delivery efficiencies of six home nebulizers using four breathing patterns. It was difficult to compare the delivery efficiencies of nebulizers because of their different residual volumes and output rates. In this study, we devised a method for quantitatively determining drug delivery efficiencies using defined variables, that is, DD/LD and DD/ED, for four breathing patterns. Finally, our results show that output rates and breathing patterns should be considered to properly determine nebulizer drug delivery efficiencies.

## Methods

### Development of the respiratory simulator

**Fig. 4:**
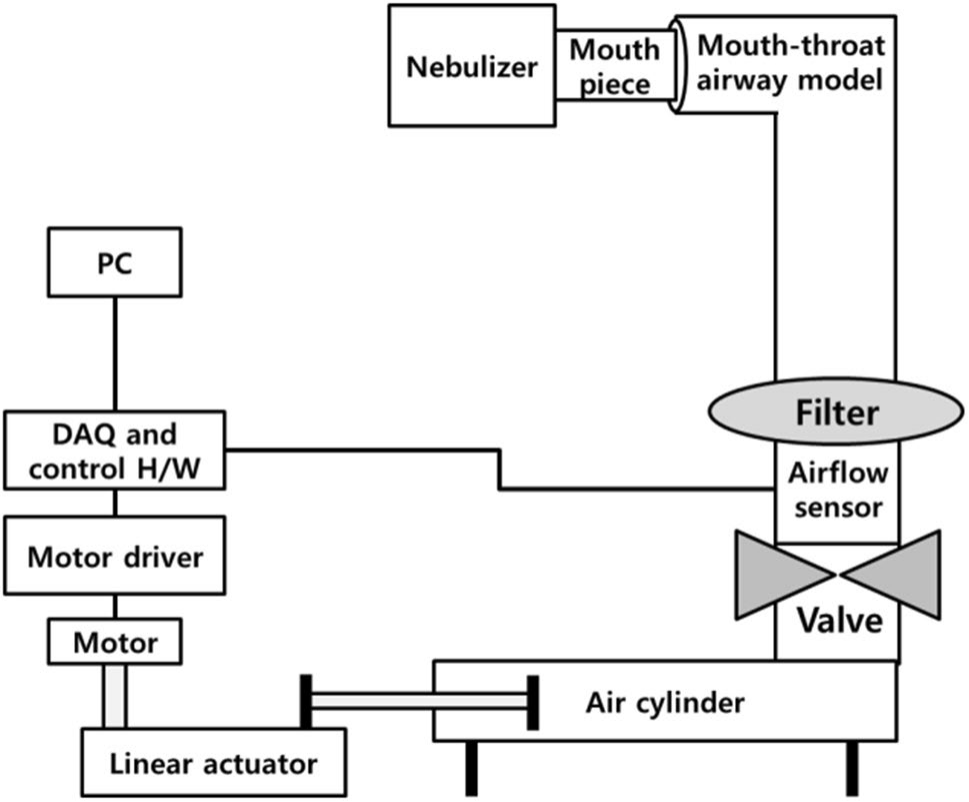
Schematic of the developed respiratory simulator

The respiratory simulator consists of a linear actuator (Scipia, Gwangju, Korea) driven by a stepper motor (Nema 57 56 Stepper motor, JingJiang Nair Motion Technologies Co., Jingjiang, China), a motor driver (TB6600, DFRobot, Shanghai, China), a flow sensor (SMF3000, Sensirion, Stafa, Switzerland), an air cylinder (MDB1B50-600, SMC Co., Tokyo, Japan), and a mouth-throat airway model. This airway model was based on airway dimensions of the oral cavity and laryngeal tracheal airway by Cheng et al., and was produced using a 3D printer (ProJet 3510HD, 3D Systems, South Carolina, Colorado, USA) and UV-curable resin (VisiJet M3, Crystal, 3D Systems, South Carolina, Colorado, USA) [39]. A disposable filter (Proguard-EX, GMS Korea, Bucheon, Korea) was located between the mouth-throat airway model and the airflow sensor. For respiration pattern control and flow data acquisition (DAQ), we used an open-source platform Arduino Uno (Arduino.cc, Ivrea, Italy). A schematic of the respiratory simulator is provided in Fig. 4.

### Calibration of the respiratory simulator

**Fig. 5:**
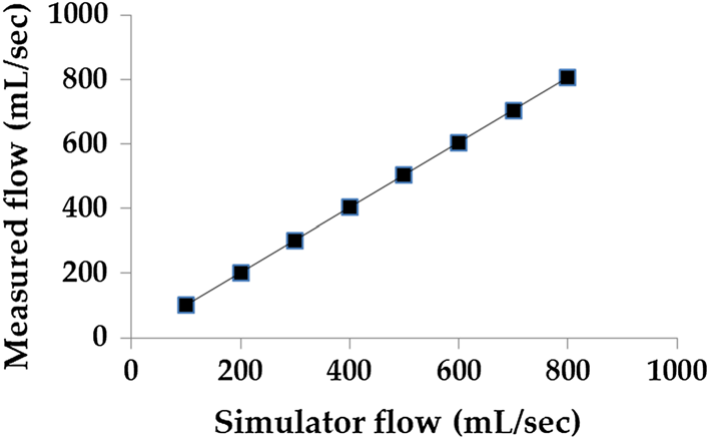
Flow calibration curve of the respiratory simulator

Initially, the air volume generated by the simulator was calibrated from 100 to 800 mL at 100 mL intervals. Tidal volume is ~ 500 mL for healthy adult males and ~ 400 mL for adult females [40]. Flows of 100 to 800 mL/s were generated and measured using a flow sensor. Data measurement was conducted at a sampling rate of 100 Hz with 10 Hz lowpass filtering. Noise removal and calculations were conducted using MATLAB (MATLAB R2018a, The MathWorks, Inc., Natick, MA, USA). The average flow error was 0.70 $\pm$ 0.12% (Fig. 5).

### Breathing patterns

Breathing patterns generated were based on ISO 27427:2013 [24], and normal adult [18], asthma [18], and COPD [27] patterns (Table 3). The respiratory simulator can be used to reproduce breathing patterns other than those mentioned in Table 3. Drug deliveries for the four breathing patterns were compared.

**Table 3:** Breathing patterns generated by the respiratory simulator

Breathing pattern	Inspiration:expiration (I:E) ratio	Respiration rate (bpm)	Tidal volume (mL)
ISO 27427:2013	1:1	15	500
Normal adult	1:2	15	500
Asthma	1:2.5	22	290
COPD	1.3	15	500

### Drug assay

Salbutamol (Ventolin respirator solution, 5 mg/mL salbutamol sulfate) was purchased from GlaxoSmithKline (Brentford, UK). A salbutamol solution of 0.1% [M/V] was prepared by diluting the supplied solution with normal saline to a concentration of 1 mg/mL. Salbutamol were determined by UV spectrophotometry at 276 nm using a SpectraMax Plus 384 (Molecular Devices, Sunnyvale, California, USA).

### Nebulizers

Six home nebulizers were tested. Modes of operation and abbreviations are provided in Table 4. PARI BOY SX + LC SPRINT was used with two nozzles (red and blue), which were supplied by the manufacturer.

**Table 4:** Tested nebulizers

Modes of operation	Models	Abbreviations used
Jet	PARI BOY SX + LC SPRINT—red nozzle (PARI GmbH, Starnberg, Germany)	JN-PARIr
	PARI BOY SX + LC SPRINT—blue nozzle (PARI GmbH, Starnberg, Germany)	JN-PARIb
Static mesh	NE-U22 (Omron Healthcare, Kyoto, Japan)	SMN-U22
	NE-U150 (Omron Healthcare, Kyoto, Japan)	SMN-U150
Vibrating mesh	NE-SM1 NEPLUS (KTMED Co., Seoul, Korea)	VMN-SM1
	NE-SM3 (KTMED Co., Seoul, Korea)	VMN-SM3

Nebulizers were charged with 2 mL of 0.1% (1 mg/mL) salbutamol as required by ISO 27427:2013 and residual volumes, nebulization times, output rates, and particle sizes were measured. Nebulizers were operated until dryness or to onset of sputter. Nebulizers were weighed before and after nebulization. Volumes were determined gravimetrically and evaporation was negligible [41]. JN-PARIb had the largest residual volume and VMN-SM1 the smallest. JN-PARIr had the longest nebulization time and SMN-U150 the shortest, and the output rate of SMN-U150 was greatest and that of JN-PARIr smallest (Table 5). Output rates were calculated as follows [3, 36].

1 $$\text{Output rate (mL/min)} = \frac{\text{charged volume (mL)}-\text{residual volume (mL)}}{\text{duration of nebulization (min)}}$$

The particle sizes (mass median diameters (MMDs)) were measured using a Spraytec (Malvern Instruments, Malvern, UK), which utilizes a laser diffraction method [42]. Fifty percent volume diameter (Dv(50)) results were automatically calculated using Spraytec software (Model #STP5311, Version 3.1, Malvern instrument, Malvern, UK, 2009). The particle size of SMN-U22 was largest and that of JN-PARIr was smallest. Results are shown in Table 5.

**Table 5:** Residual volumes, nebulization times, output rates, and MMDs (Dv(50)) values of the six nebulizers

Device	Residual volume (mL)	Nebulization time (min)	Output rate (mL/min)	MMDs (Dv(50)) (${\upmu }\hbox{m}$)
JN-PARIr	0.539 $\pm$ 0.020	8.13 $\pm$ 0.10	0.182 $\pm$ 0.002	3.55 $\pm$ 0.10
JN-PARIb	0.629 $\pm$ 0.034	6.07 $\pm$ 0.23	0.231 $\pm$ 0.003	4.91 $\pm$ 0.09
SMN-U22	0.096 $\pm$ 0.038	6.22 $\pm$ 0.21	0.307 $\pm$ 0.015	7.14 $\pm$ 0.08
SMN-U150	0.421 $\pm$ 0.011	4.81 $\pm$ 0.30	0.334 $\pm$ 0.023	6.43 $\pm$ 0.09
VMN-SM1	0.009 $\pm$ 0.005	7.93 $\pm$ 0.64	0.254 $\pm$ 0.022	5.09 $\pm$ 0.11
VMN-SM3	0.015 $\pm$ 0.004	6.09 $\pm$ 0.14	0.329 $\pm$ 0.007	7.06 $\pm$ 0.16

### Delivery efficiency experiments

Amounts of drug delivered were measured for nebulization durations and at constant output (Table 6). To calculate the percentages of delivered doses, amounts collected by the filter were expressed as percentages of the amounts of salbutamol loaded in nebulizer reservoirs (DD/LD) or as percentages of emitted doses (DD/ED). The loading dose (LD) in nebulizer reservoirs was set at 2 mg for Experiments 1 and 2 and the emitted dose (ED) was defined as nebulizer drug output.

- Experiment 1: Nebulizers were operated for duration of nebulization (until dryness or the onset of sputter).
- Experiment 2: Nebulizers were operated at constant output (1.3 mL).

Experiment 1 represented a common clinical condition, but doses emitted by nebulizers vary, and it is difficult to evaluate percentage lung delivered efficiencies with respect to emitted doses. The JN-PARIb nebulizer had the largest residual volume (~ 0.7 mL), and 1.3 mL was chosen as the constant output for Experiment 2.

**Table 6:** Test conditions for the delivery of 0.1% salbutamol (1 mg/mL)

Experiment	Loading dose (mg)	Operating conditions
1	2	Duration of nebulization
2	2	Constant output 1.3 mL

The amounts of delivered drug collected by filters were quantified by UV spectrophotometry. Salbutamol collected in filters was eluted with distilled water (15 mL) for 24 h. Delivery efficiency percentages were calculated with respect to amounts of salbutamol loaded or emitted (DD/LD and DD/ED, respectively). DD/LD values were calculated using the following formula.

2 $$\text{Percentage delivered dosage for loading dose (DD/LD)}(\%) = \frac{\text{lung delivered dose (mg)}}{\text{loading dose (mg)}}$$

DD/LD values were calculated using a loading dose of 2 mg, and thus, larger residual volumes resulted in lower delivery efficiencies. Thus, to compare delivery efficiencies for emitted doses (ED), we calculated DD/ED values using the following formula.

3 $$\text{Percentage delivered dosage for emitted dose (DD/ED)}(\%) = \frac{\text{lung delivered dose (mg)}}{\text{emitted dose (mg)}}$$

In order to compare the drug delivery efficiencies of nebulizers for each breathing pattern, delivered doses and percentage delivered dosages were compared versus those obtained for the ISO 27427:2013 breathing pattern.

### Statistical analysis

Correlations between simulated and measured respiration air flows were evaluated using Spearman’s correlation coefficients. Lung delivered doses are presented as means and standard deviations. Data were analyzed by two-way analysis of variance followed by Dunnett’s post hoc test. The analysis was performed using R-studio Version 4.0.3 (R-studio, MA, USA, 2020), and *p* values of $< 0.05$ were considered significant.

## Data Availability

Not applicable.

## Abbreviations

JN-PARIr: PARI BOY SX + LC SPRINT—red nozzle (PARI GmbH, Starnberg, Germany)
JN-PARIb: PARI BOY SX + LC SPRINT—blue nozzle (PARI GmbH, Starnberg, Germany)
SMN-U150: NE-U150 (Omron Healthcare, Kyoto, Japan)
SMN-U22: NE-U22 (Omron Healthcare, Kyoto, Japan)
VMN-SM1: NE-SM1 NEPLUS (KTMED Co., Seoul, Korea)
VMN-SM3: NE-SM3 (KTMED Co., Seoul, Korea).
